# The Synthesis and Antiproliferative Activities of New Arylidene-Hydrazinyl-Thiazole Derivatives

**DOI:** 10.3390/ijms151222059

**Published:** 2014-12-01

**Authors:** Adriana Grozav, Luiza Ioana Găină, Valentina Pileczki, Ovidiu Crisan, Luminita Silaghi-Dumitrescu, Bruno Therrien, Valentin Zaharia, Ioana Berindan-Neagoe

**Affiliations:** 1 Faculty of Pharmacy, “Iuliu Hatieganu” University of Medicine and Pharmacy, Victor Babes 41, RO-400012 Cluj-Napoca, Romania; E-Mails: ocrisan@umfcluj.ro (O.C.); vzaharia@umfcluj.ro (V.Z.); 2 Faculty of Chemistry and Chemical Engineering, University “Babes-Bolyai”, M. Kogalniceanu 1, RO-400028 Cluj-Napoca; E-Mail: lusi@chem.ubbcluj.ro; 3 Research Center of Functional Genomics, Biomedicine and Translational Medicine, “Iuliu Hatieganu” University of Medicine and Pharmacy, Marinescu 23, RO-400337 Cluj-Napoca, Romania; E-Mails: valentinapilecki@gmail.com (V.P.); ioananeagoe29@gmail.com (I.B.-N.); 4 Institut de Chimie, Université de Neuchâtel, Avenue de Bellevaux 51, CH-2000 Neuchâtel, Switzerland; E-Mail: bruno.therrien@unine.ch; 5 Department of Immunology, “Iuliu Hatieganu” University of Medicine and Pharmacy, Victor Babes 8, RO-400012 Cluj-Napoca, Romania

**Keywords:** thiazole, hydrazine, cytotoxicity, DNA interaction

## Abstract

New and known arylidene-hydrazinyl-thiazole derivatives have been synthesized by a convenient Hantzsch condensation. All compounds were evaluated for their *in vitro* cytotoxicity on two carcinoma cell lines, MDA-MB231 and HeLa. Significant antiproliferative activity for 2-(2-benzyliden-hydrazinyl)-4-methylthiazole on both MDA-MB-231 (IC_50_: 3.92 µg/mL) and HeLa (IC_50_: 11.4 µg/mL) cell lines, and for 2-[2-(4-methoxybenzylidene) hydrazinyl]-4-phenylthiazole on HeLa (IC_50_: 11.1 µg/mL) cell line is reported. Electrophoresis experiments showed no plasmid DNA (pTZ57R) cleavage in the presence of the investigated thiazoles.

## 1. Introduction

Recent studies have shown a constant interest in thiazole compounds due to a wide spectra of biologic activities, such as the antimalarial activity of hydrazinyl-thiazoles [1], antiproliferative activity of steroidal[17,16-d]thiazole against gastric carcinoma cells [2], antitumor activity of thiazol-1*H*-pyrrolo-[2,3-b]pyridine in peritoneal mesothelioma experimental models [3], antiproliferative activity of thiazol-1H-indoles and thiazol-1H-7-azaindoles in MiaPaCa-2 cell line [4], CDK-1 inhibitory activity of thiazol-1H-pyrrolo[3,2-b]pyridine [5], antimicrobial activity of thiazole-oxadiazole derivatives [6], or anti-inflammatory properties of hydrazono-thiazole derivatives [7]. The thiazole rings can be found in a variety of pharmaceutical drugs, such as Ritonavir (anti-HIV) [8], Bleomycin [9] and Tiazofurin (antineoplastics) [10], Fanetizole and Meloxicam (anti-inflammatories) [11], which explains the interest in the development of new compounds containing this heterocyclic unit.

Regarding the synthesis of hydrazinyl-thiazoles, two procedures have been highlighted in the literature: the classical condensation of a carbonyl group with thiosemicarbazide followed by the cyclization of thiosemicarbazones with α-halocarbonyl derivatives [12,13,14,15], and a more recently reported one-step multi-component synthetic protocol [16,17].

The main goal of this work was to identify new possible chemotherapeutic agents based on organic heterocyclic derivatives, which are less harmful for the human body than the well-known platinum derivatives. In this paper, we present a two-step protocol for the synthesis of seven new arylidene-hydrazinyl-thiazoles **2c**, **2f**, **2h**, **2j**, **2l**, **2m**, **2p** and nine previously reported thiazoles **2a**, **2b**, **2d**, **2e**, **2g**, **2i**, **2k**, **2n**, **2o**, followed by the *in vitro* evaluation of the antiproliferative activity on two carcinoma cell lines, MDA-MB231 and HeLa. To identify a possible correlation between DNA damage and cytotoxicity, the interaction of the thiazole derivatives **2a**, **2e**, **2h**, **2i** with DNA was evaluated by electrophoresis.

## 2. Results and Discussion

### 2.1. Synthesis of Arylidene-Hydrazinyl-Thiazole Derivatives 2a–p

**Scheme 1:**
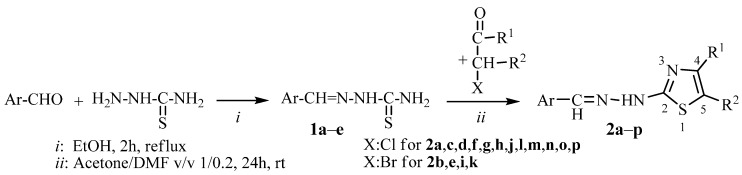
Synthesis of arylidene-hydrazinyl-thiazoles **2a**–**p**.

A series of arylidene-hydrazinyl-thiazole derivatives **2a**–**p** were synthesized in two steps: the condensation of aromatic aldehydes with hydrazinecarbothioamide, followed by the cyclization of aryliden-thiosemicarbazones **1a**–**e** with α-halocarbonyl derivatives (Scheme 1, Table 1). Both the condensation and cyclization reactions were performed in good yield by the Hantzsch protocol. Derivatives **2a**, **2b**, **2d**, **2e**, **2g**, **2i**, **2k**, **2n** and **2o** have been previously prepared by other groups [17,18,19,20].

**Table 1:** Functional groups of the hydrazinyl-thiazole derivatives **2a**–**p**.

**Compound 1**	**a**	**b**	**c**	**d**	**e**			
Ar	C_6_H_5_	C_6_H_3_Cl_2_(2,4)	C_6_H_4_OH(4)	C_6_H_4_OCH_3_(4)	C_6_H_4_Cl(3)			
**Compodund 2**	**a**	**b**	**c**	**d**	**e**	**f**	**g**	**h**
Ar	C_6_H_5_	C_6_H_5_	C_6_H_5_	C_6_H_4_OCH_3_(4)	C_6_H_4_OCH_3_(4)	C_6_H_4_OCH_3_(4)	C_6_H_4_OCH_3_(4)	C_6_H_4_OH(4)
R^1^	CH_3_	C_6_H_5_	CH_3_	CH_3_	C_6_H_5_	CH_3_	CH_3_	CH_3_
R^2^	H	H	COCH_3_	H	H	COCH_3_	COOC_2_H_5_	H
**Compound 2**	**i**	**j**	**k**	**l**	**m**	**n**	**o**	**p**
Ar	C_6_H_4_OH(4)	C_6_H_4_Cl(3)	C_6_H_4_Cl(3)	C_6_H_4_Cl(3)	C_6_H_4_Cl(3)	C_6_H_3_Cl_2_(2,4)	C_6_H_3_Cl_2_(2,4)	C_6_H_3_Cl_2_(2,4)
R^1^	C_6_H_5_	CH_3_	C_6_H_5_	CH_3_	CH_3_	CH_3_	CH_3_	CH_2_COOC_2_H_5_
R^2^	H	H	H	COCH_3_	COOC_2_H_5_	COCH_3_	COOC_2_H_5_	H

NMR and MS spectra were recorded for all the arylidene-hydrazinyl-thiazoles **2a**–**p**. The ^1^H-NMR spectra of arylidene-hydrazinyl-thiazoles **2a**–**p** present a similar pattern for the hydrazone unit. The most downfield singlet, around 12 ppm, corresponds to the hydrazinyl moiety (N-NH), which is only present in DMSO-*d*_6_ solutions, and otherwise missing due to the deuterium exchange. The singlet around 8.4~7.8 ppm is assigned to the azomethine proton (CH=N). The expected molecular ion (M^+^) is found in the mass spectra of all arylidene-hydrazinyl-thiazoles **2a**–**p**. Moreover, the fragmentation pathway involved the cleavage of the nitrogen-nitrogen bond from the hydrazinyl unit. For example, in the MS spectra of thiazole **2a**, this fragmentation generates a peak at *m*/*z* 113 for the aza-thiazole ion, while for the thiazole **2b** the corresponding aza-thiazole ion peak is observed at *m*/*z* 175, in accordance with the substitution of the thiazole heterocycle.

### 2.2. In Vitro Cytotoxicity Assay

The anti-proliferative activity of the sixteen arylidene-hydrazinyl-thiazole derivatives against two human carcinoma MDA-MB231 and HeLa cell lines was evaluated using MTT assays [14,21] after 24 h of treatment. According to the IC_50_ data (Table 2), five thiazole derivatives, **2a**, **2e**, **2f**, **2h** and **2i**, have shown significant inhibition on both MDA-MB231 and HeLa cancer cell lines. Their activity is comparable or even better than that of the platinum drugs cisplatin and oxaliplatin, which were used as controls.

Having the IC_50_ values for thiazoles **2a**–**p**, we tried to establish a correlation between the cytotoxic activity and the molecular structure, by looking at the nature of the functional groups and their position on the arylidene-hydrazinyl-thiazole backbone. The presence of a methyl or phenyl group in position 4 (see Scheme 1) and a hydrogen or acetyl in position 5 on the thiazole ring, combined with phenyl, *p*-OH-phenyl or *p*-MeO-phenyl as the aromatic group attached to the hydrazinyl unit, led to compounds **2a**, **2e**, **2f**, **2h** and **2i**, which exhibited the highest antiproliferative activity. On the other hand, the presence of chlorine atoms at the phenyl hydrazinyl unit and ethyl carboxylate group in position 5 on the thiazole ring **2m**–**o** decreased the antiproliferative efficiency (Table 2).

**Table 2:** IC_50_ values for thiazoles **2a**–**p** on the MDA-MB231 and HeLa cell lines.

Compound	IC50 (µg/mL)	
	MDA-MB-231	HeLa
**2a**	3.92 ± 0.015	11.4 ± 0.005
**2b**	35.5 ± 0.003	>100
**2c**	>100	64.87 ± 0.005
**2d**	>100	>100
**2e**	46.11 ± 0.009	11.1 ± 0.009
**2f**	16.25 ± 0.008	>100
**2g**	>100	>100
**2h**	48.44 ± 0.017	25.59 ± 0.010
**2i**	18.54 ± 0.008	20.04 ± 0.019
**2j**	>100	57.53 ± 0.011
**2k**	81.02 ± 0.001	>100
**2l**	75.50 ± 0.009	>100
**2m**	>100	>100
**2n**	>100	>100
**2o**	>100	>100
**2p**	64.95 ± 0.009	>100
Cisplatin	17.28 ± 0.002	26.12 ± 0.010
Oxaliplatin	14.09 ± 0.001	23.17± 0.011

**Figure 1:**
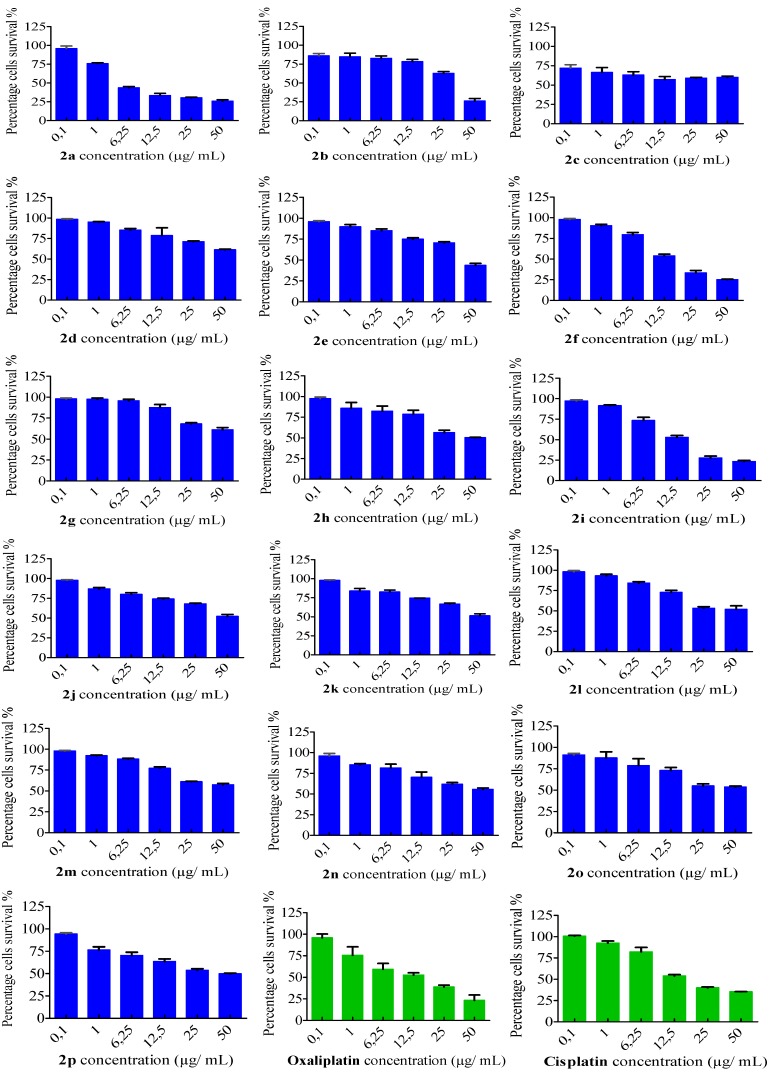
Viability effects of arylidene-hydrazinyl-thiazoles **2a**–**p** on MDA-MB-231 cells using the MTT assay.

**Figure 2:**
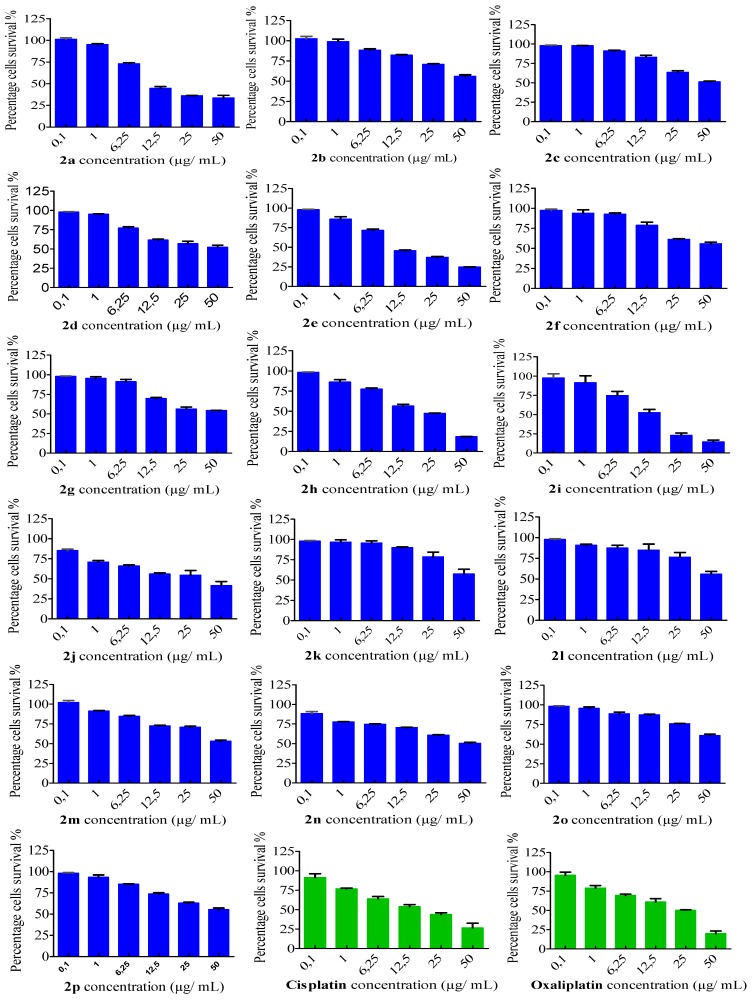
Viability effects of arylidene-hydrazinyl-thiazoles **2a**–**p** on HeLa cells using the MTT assay.

The viability of the breast cancer MDA-MB-231 cells and cervical cancer HeLa cells decreased with an increase in the concentration of the thiazole derivatives **2a**–**p** (Figure 1 and Figure 2). The profiles of the MDA-MB-231 cells survival viability, correlated to the thiazole doses, revealed a common trend for thiazoles **2a**, **2f**, **2i**, as well as cisplatin and oxaliplatin (Figure 1). For the HeLa cell line, the same correlation is observed between compounds **2a**, **2e**, **2h**, **2i** and the chemotherapeutic drugs cisplatin and oxaliplatin (Figure 2).

Due to its significant antiproliferative activity on both MDA-MB-231 (IC_50_: 3.92 µg/mL) and HeLa (IC_50_: 11.4 µg/mL) cell lines, the 2-(2-benzyliden-hydrazinyl)-4-methylthiazole derivative **2a** was studied further and used as a starting point for the development of new arylidene-hydrazinyl-thiazole compounds for the treatment of cancer. Additionally, 2-[2-(4-methoxybenzylidene) hydrazinyl]-4-phenylthiazole (**2e**), with an IC_50_ value of 11.1 µg/mL, was also considered as a potentially useful cytotoxic compound against the HeLa cell line.

### 2.3. DNA Intercalation Study

**Figure 3:**
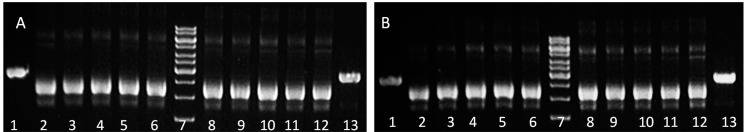
Interaction of thiazoles **2a**, **2e**, **2h** and **2i** with plasmid DNA. (**A**) compounds **2a** (lines 2–6) and **2h** (lines 8–12); (**B**) compounds **2i** (lines 2–6) and **2e** (lines 8–12). The order was the same in all cases. Lane 1: linear plasmid (plasmid DNA digested with restriction enzyme *Eco*RI); 2: closed circular plasmid DNA; 3 to 6: closed circular plasmid DNA with 2, 4, 8 and 10 μL of the compound; 7: GeneRuler 1 kb DNA ladder (ThermoScientific), 8: closed circular plasmid DNA; 9 to 12: closed circular plasmid DNA with 2, 4, 8 and 10 μL of the compound; 13: linear plasmid.

For the best candidates, the arylidene**-**hydrazine**-**thiazole derivatives **2a**, **2e**, **2h** and **2i**, their interactions with plasmid DNA (pTZ57R) were investigated. Gel electrophoresis experiments investigating the plasmid migration in agarose gel after incubation with thiazoles **2a**, **2e**, **2h** and **2i** did not reveal any ability of these compounds to generate changes in the electrophoretic mobility of supercoiled DNA. These electrophoresis experiments showed that DNA cleavage does not occur in the presence of thiazoles **2a**, **2e**, **2h** and **2i**, even at elevated concentrations of the selected compounds (Figure 3).

The gel electrophoresis results for the pTZ57R DNA incubated with thiazole derivatives **2a**, **2e**, **2h** and **2i** suggested that, despite increasing thiazole concentrations, no meaningful effect on plasmid DNA was observed. This is an indication that the cytotoxic effect of these derivatives against MDA-MB-231 and HeLa cell lines does not involve interaction with DNA.

## 3. Experimental Section

### 3.1. Materials and Methods

The starting materials and solvents were obtained from commercial sources. The reagents used for cell culture experiments (fetal calf serum (FCS), penicillin-streptomycin, glutamine and RPMI 1460 cell media) were purchased from Sigma-Aldrich (St. Louis, MO, USA). The antineoplastic drugs cisplatin and oxaliplatin were purchased from Actavis Sindan Pharma (Bucharest, Romania). The GeneRuler 1 kb DNA ladder was purchased from Thermo Scientific (Waltham, MA, USA).

Compounds **1a**–**e** were prepared according to the literature [14]. Melting points were measured with an Electrothermal IA 9200 apparatus (Bibby Scientific Limited (Group HQ), Stone, UK), and are uncorrected values. ^1^H-NMR and ^13^C-NMR spectra were recorded in CDCl_3_, acetone-*d*_6_ or DMSO-*d*_6_ (locked to Me_4_Si) using a 400 or 600 MHz Bruker Avance NMR spectrometer (Bruker Biospin GmbH, Rheinsberg, Germany). Elemental analysis was carried out on a Vario EL III instrument. The mass spectra were recorded with a Shimadzu QP 2010 Plus GC-MS instrument (Shimadzu Corporation, Kyoto, Japan) and a Thermo Scientific LTQ Orbitrap XL mass spectrometer (Thermo Fisher Scientific Inc., Pittsburgh, PA, USA).

The equipment involved in cell lines multiplication included Class II LaminAir laminar hoods, a ShelLab incubator, and an Eppendorf Centrifuge 5702R with spin-out rotor. Spectrophotometric measurements were completed with Biotek Synergy 2 Multi-Mode Microplate Reader with SQ Xenon Flash light source, using well-area colorimetric scanning.

The MTT experimental data were processed with Graph Pad Prism 5 biostatistics software (Sheldon Manufacturing, Inc., Cornelius, OR, USA).

For the cytotoxicity assessment, two highly proliferative MDA-MB-231 and HeLa tumor cells were utilized. Both cell lines used in the experiment were purchased from the European Collection of Cell Cultures (ECCAC, BioTek, Winooski, VT, USA).

### 3.2. General Procedure for the Synthesis of Compounds 2a–p

A mixture of arylidene-hydrazine-carbothioamide (10 mmol) and α-halogenocarbonyl derivative (10 mmol) in acetone/DMF (10 mL, *v*/*v*: 1/0.2) was stirred at room temperature for 20–24 h; the reaction progress was monitored by TLC (toluene/AcOEt 2/1 *v*/*v*; silica plates). The reaction mixture was neutralized at pH 7 with NaHCO_3_ aqueous solution (10%). The precipitate was filtered and recrystallized from ethanol or acetic acid. For all compounds the yield, the melting point, the EI MS, and the elemental analysis are given, while the ^1^H- and ^13^C-NMR data are only provided for the new derivatives.

**2a**. (*E*)-2-(2-Benzylidenehydrazinyl)-4-methylthiazole [18]: White crystals, yield 1.7 g, 78%, m.p. 190–191 °C, crystallized from ethanol (m.p. lit. 192–194 °C); EI *m*/*z*: 218 (M^+^), 113 (100%), 77; Calcd. for C_11_H_11_N_3_S: C, 59.09; H, 4.46; N, 20.67; Found: C, 59.11; H, 4.49; N, 20.65.

**2b**. (*E*)-2-(2-Benzylidenehydrazinyl)-4-phenylthiazole [19]: Light brown crystals, yield 2.3 g, 81%, m.p. 218–219 °C, crystallized from ethanol (m.p. lit. 220 °C); EI *m*/*z*: 279 (M^+^), 176 (100%), 104, 77; Calcd. for C_16_H_13_N_3_S: C, 68.79; H, 4.69; N, 15.04; Found: C, 68.82; H, 4.71; N, 15.02.

**2c**. (*E*)-1-(2-(2-(Benzylidene)hydrazinyl)-4-methylthiazol-5-yl)ethanone: Yellow crystals, yield 1.9 g, 76%, m.p. 222–223 °C, crystallized from ethanol; ^1^H-NMR (DMSO-*d*_6_, 400 MHz, δ ppm): 2.41 (s, 3H), 2.51 (s, 3H), 7.47–7.49 (m, 3H), 7.69 (dd, 2H, ^3^*J* = 7.8Hz, ^4^*J* = 1.3Hz), 8.11 (s, 1H), 12.48 (s, 1H); ^13^C-NMR (DMSO-*d*_6_, 100 MHz, δ ppm): 18.1, 29.5, 122.4, 126.7 (2C), 128.8 (2C), 129.8, 134.1, 144.9, 156.6, 169.1, 188.8; EI *m*/*z*: 259 (M^+^), 182, 141 (100%), 77; Calcd. for C_13_H_13_N_3_OS: C, 60.21; H, 5.05; N, 16.20; Found: C, 60.24; H, 5.09; N, 16.19.

**2d**. (*E*)-2-(2-(4-Methoxybenzylidene)hydrazinyl)-4-methylthiazole [19]: White crystals, yield 1.6 g, 68%, m.p. 179–180 °C, crystallized from ethanol (m.p. lit. 170 °C); EI *m*/*z*: 247 (M^+^), 140, 134, 114 (100%), 107, 77; Calcd. for C_12_H_13_N_3_OS: C, 58.28; H, 5.30; N, 16.99; Found: C, 58.31; H, 5.33; N, 16.97.

**2e**. (*E*)-2-(2-(4-Methoxybenzylidene)hydrazinyl)-4-phenylthiazole [19]: Light orange crystals, yield 2.3 g, 76%, m.p. 195–196 °C, crystallized from ethanol (m.p. lit. 196 °C); EI *m*/*z*: 309 (M^+^), 202, 176 (100%), 133, 107, 77; Calcd. for C_17_H_15_N_3_OS: C, 66.00; H, 4.89; N, 13.58; Found: C, 66.04; H, 4.92; N, 13.57.

**2f**. (*E*)-1-(2-(2-(4-Methoxybenzylidene)hydrazinyl)-4-methylthiazol-5-yl)ethanone: White yellow crystals, yield 2.1 g, 73%, m.p. 214–215 °C, crystallized from ethanol; ^1^H-NMR (DMSO-*d*_6_, 400 MHz, δ ppm): 2.41 (s, 3H), 2.49 (s, 3H), 3.80 (s, 3H), 7.01 (d, 2H, ^3^*J* = 8.6 Hz), 7.64 (d, 2H, ^3^*J* = 8.6 Hz), 8.06 (s, 1H), 12.27 (s, 1H); ^13^C-NMR (DMSO-*d*_6_, 100 MHz, δ ppm): 17.5, 29.1, 55.3, 114.3 (2C), 112.8, 126.5, 128.3 (2C), 144.9, 156.7, 160.7, 169.1, 188.3; EI *m*/*z*: 289 (M^+^), 141 (100%), 134, 120, 107, 77; Calcd. for C_14_H_15_N_3_O_2_S: C, 58.11; H, 5.23; N, 14.52; Found: C, 58.14; H, 5.27; N, 14.53.

**2g**. (*E*)-Ethyl 2-(2-(4-methoxybenzylidene)hydrazinyl)-4-methylthiazole-5-carboxylate [20]: White crystals, yield 2.5 g, 81%, m.p. 181–182 °C, crystallized from ethanol, (m.p. lit. 180–182 °C); EI *m*/*z*: 319 (M^+^), 186 (100%), 134, 107, 77; Calcd. for C_15_H_17_N_3_O_3_S: C, 56.41; H, 5.37; N, 13.16; Found: C, 56.45; H, 5.40; N, 13.17.

**2h**. (*E*)-4-((2-(4-Methylthiazol-2-yl)hydrazono)methyl)phenol: Brown crystals, yield 0.9 g, 42%, m.p. 196 °C, crystallized from acetic acid; ^1^H-NMR (acetone-*d*_6_, 600 MHz, δ ppm): 2.16 (s, 3H), 4.28 (bb, 1H), 6.28 (s, 1H), 6.87 (d, 2H, ^3^*J* = 8.6Hz), 7.52 (d, 2H, ^3^*J* = 8.6Hz), 7.97 (s, 1H); ^13^C-NMR (acetone-*d*_6_, 125 MHz, δ ppm): 16.9, 102.8, 116.4 (2C), 126.9, 128.9 (2C), 143.3, 148.0, 169.9, 173.2; EI *m*/*z*: 233 (M^+^), 120, 114 (100%), 107, 77; Calcd. for: C_11_H_11_N_3_OS, C, 56.63; H, 4.75; N 18.01; Found: C, 56.65; H, 4.79; N, 18.03.

**2i**. (*E*)-4-((2-(4-Phenylthiazol-2-yl)hydrazono)methyl)phenol [22]: Light orange crystals, yield 1.4 g, 48%, m.p. 242 °C, crystallized from acetic acid, (m.p. lit. 241–243 °C); EI *m*/*z*: 295 (M^+^), 202, 176 (100%), 134, 120, 77; Calcd. for: C_16_H_13_N_3_OS; C, 65.06; H, 4.44; N, 14.23; Found: C, 65.10; H, 4.47; N, 14.20.

**2j**. (*E*)-2-(2-(3-Chlorobenzylidene)hydrazinyl)-4-methylthiazole: White crystals, yield 1.5 g, 69%, m.p. 177–178 °C, crystallized from ethanol; ^1^H-NMR (CDCl_3_, 600 MHz, δ ppm): 2.33 (s, 3H), 6.22 (s, 1H), 7.31 (d, 2H, ^3^J = 7.2Hz), 7.48 (t, 1H, ^3^J = 7.2 Hz), 7.66 (s, 1H), 7.82 (s, 1H); ^13^C-NMR (DMSO-d_6_, 125 MHz, δ ppm): 16.9, 102.5, 124.8, 125.3, 128.6, 130.6, 133.6, 136.9, 139.6, 146.7, 167.9. EI m/z: 251/253 (M^+^/M^+2^), 140, 138, 114 (100%), 111; Calcd. for: C_11_H_10_ClN_3_S; C, 52.48; H, 4.00; N, 16.69; Found: C, 52.52; H, 4.03; N, 16.67.

**2k**. (*E*)-2-(2-(3-Chlorobenzylidene)hydrazinyl)-4-phenylthiazole [17]: White green crystals, yield 2.2 g, 72%, m.p. 183–184 °C, crystallized from ethanol, (m.p. lit. 163–164 °C); EI *m*/*z*: 313/315 (M^+^/M^+2^), 202, 176 (100%), 138, 111; Calcd. for: C_16_H_12_ClN_3_S: C, 61.24; H, 3.85; N, 13.39; Found: C, 61.28; H, 3.88; N, 13.37.

**2l**. (*E*)-1-(2-(2-(3-Chlorobenzylidene)hydrazinyl)-4-methylthiazol-5-yl)ethanone: Light yellow crystals; yield: 2 g, 71%, m.p. 239–240 °C crystallized from ethanol; ^1^H-NMR (DMSO-d_6_, 400 MHz, δ ppm): 2.41 (s, 3H), 2.49 (s, 3H), 7.44–7.47 (m, 2H, ^3^J = 7.1Hz), 7.63 (t, 1H, ^3^J = 7.1Hz), 7.7 (s, 1H), 8.07 (s, 1H), 12.32 (s, 1H); ^13^C-RMN (DMSO-d_6_, 100 MHz, δ ppm): 17.9, 29.5, 121.9, 125.3, 125.9, 129.4, 130.7, 133.6, 136.2, 143.2, 169.0, 172.0, 188.0. EI m/z: 293/295 (M^+^/M^+2^), 250, 182, 156, 141 (100%), 138, 111. Calcd. for C_13_H_12_ClN_3_OS: C, 53.15; H, 4.12; N, 14.30; Found: C, 53.17; H, 4.15; N, 14.28.

**2m**. (*E*)-Ethyl 2-(2-(3-chlorobenzylidene)hydrazinyl)-4-methylthiazole-5-carboxylate: White crystals, yield 2.3 g, 72%, m.p. 235–236 °C, crystallized from ethanol; ^1^H-NMR (DMSO-d_6_, 400 MHz, δ ppm): 1.27 (t, 3H, ^3^J = 7.1 Hz), 2.47 (s, 3H), 4.21 (q, 2H, ^3^J = 7.1 Hz), 7.46–7.49 (m, 2H,), 7.67–7.65 (m, 1H), 7.72 (s, 1H), 8.09 (s, 1H), 12.56 (s, 1H); ^13^C-RMN (acetone-d_6_, 100 MHz, δ ppm): 14.6, 31.1, 60.2, 125.6, 126.4, 129.8, 131.2, 133.1, 134.9, 140.0, 156.0, 165.6, 171.1. EI m/z: 323/325 (M^+^/M^+2^), 212, 186 (100%); Calcd. for C_14_H_14_ClN_3_O_2_S: C, 51.93; H, 4.36; N, 12.98; Found: C, 51.97; H, 4.39; N, 12.96.

**2n**. (*E*)-1-(2-(2-(2,4-Dichlorobenzylidene)hydrazinyl)-4-methylthiazol-5-yl)ethanone [20]: Yellow crystals, yield 2.6 g, 81%, m.p. 241–242 °C, crystallized from ethanol, (m.p. lit. 240–242 °C); EI *m*/*z*: 327/329/ (M^+^/M^+2^), 315, 156, 141 (100%), 145, 112; Calcd. for C_13_H_11_Cl_2_N_3_OS: C, 47.57; H, 3.38; N, 12.80; Found: C, 47.60; H, 3.40; N, 12.79.

**2o**. (*E*)-Ethyl 2-(2-(2,4-dichlorobenzylidene)hydrazinyl)-4-methylthiazole-5-carboxylate [20]: White crystals, yield 2.8 g, 79%, m.p. 224–225 °C, crystallized from ethanol, (m.p. lit. 223–224 °C); EI *m*/*z*: 357/359 (M^+^/M^+2^), 212, 186 (100%), 172, 112; Calcd. for C_14_H_13_Cl_2_N_3_O_2_S: C, 46.94; H, 3.66; N, 11.73; Found: C, 46.97; H, 3.69; N, 11.71.

**2p**. (*E*)-Ethyl 2-(2-(2-(2,4-dichlorobenzylidene)hydrazinyl)thiazol-4-yl)acetate: White yellow crystals, yield 2.8 g, 80%, m.p. 140–141 °C, crystallized from ethanol; ^1^H-NMR (DMSO-*d*_6_, 400 MHz, δ ppm): 1.19 (t, 3H, ^3^*J* = 7 Hz), 3.58 (s, 2H), 4.08 (q, 2H, ^3^*J* = 7 Hz), 6.69 (s, 1H), 7.48 (dd, 1H, ^3^*J* = 8.5 Hz, ^4^*J* = 1.9 Hz), 7.65 (d, 1H, ^4^*J* = 1.9 Hz), 7.87 (d, 1H, ^3^*J* = 8.5 Hz), 8.25 (s, 1H), 12.29 (s, 1H); ^13^C-NMR (DMSO-*d*_6_, 100 MHz, δ ppm): 14.1, 36.8, 60.2, 102.2, 127.3, 127.9, 129.3, 130.7, 132.7, 133.9, 136.9, 145.6, 169.9; EI *m*/*z*: 357/359 (M^+^/M^+2^), 212, 186, 170 (100%); Anal. Calcd. for C_14_H_13_Cl_2_N_3_O_2_S: C, 46.94; H, 3.66; N, 11.73; Found: C, 46.97; H, 3.68; N, 11.71.

### 3.3. In Vitro Anticancer Screening

#### 3.3.1. Cell Cultures

Both MDA-MB-231 and HeLa cell lines were grown under sterile conditions in Cole-type culture flasks (25 cm^2^, Nunclon Easy Flask), using cell growth media (RPMI 1460) supplemented with 5% fetal calf serum (FCS), 0.1% penicillin–streptomycin, and 0.1% glutamine. The culture flasks were kept in an incubator at constant humidified atmosphere, temperature (37 °C), and CO_2_ level (5%). The cells passage was performed by enzymatic methods using Trypsin.

#### 3.3.2. Cell Treatment and Cytotoxicity Evaluation

For cytotoxicity evaluation, the stock solutions of thiazoles **2a**–**p** in DMSO (2000 μg/mL) were used to prepare diluted samples with the following concentrations: 0.1, 1, 6.25, 12.50, 25 and 50 µg/mL using RPMI 1460 media. The cells were placed on flat bottom 96-well micro plates for tissue culture (*ca.* 10.000 cells/well) and cultured in complete medium as described above (200 μL).

After 24 h, the solutions of thiazoles **2a**–**p** were added separately to each well.

After treatment (24 h) with thiazole derivatives, the culture medium was removed from the wells, without disturbing the attached cells, and 60 μL of MTT-Hanks media solution was added to each well. After incubating the plates for 2 h at 37 °C, the MTT solution was removed, and the formazan crystals were solubilized by adding DMSO (100 μL).

The 96-well plates were measured with a multimode microplate reader, by monochromator-based absorbance detection at 570 nm wavelength. The optical density, quantified by colorimetric measurements, is directly proportional with the amount of formazan crystals formed in the cells and it is an indicator of the cellular viability.

Untreated cells were used as reference for cell proliferation. For each compound, reagent blank (media and MTT) and color control (wells containing media and thiazole derivatives solution, without cells) were used. For Positive control, two antineoplastic drugs, cisplatin and oxaliplatin, were used in the same concentrations as the studied compounds. All experiments were performed in triplicate.

### 3.4. DNA Electrophoresis Tests

The plasmid DNA was purified from an overnight culture of *Escherichia coli* DH5α cells using the DNA-spin™ Plasmid DNA Purification Kit (ThermoScientific).

The DMSO solutions of thiazoles **2a**, **2e**, **2h** (1 mM) and **2i** (2 mM) were mixed in different ratios (2, 4, 8, 10 µL) with closed circular plasmid DNA pTZ57R, (1 µL, 540 ng), resulting in nucleotide/thiazoles ratios of 1:1.2, 1:2.6, 1:1.5, 1:6.5 for compounds **2a**, **2h**, **2e** and 1:2.6; 1:5.2; 1:10; 1:13 for compound **2i**. The mixtures were loaded into agarose gel 1% in TAE buffer (40 mM tris-acetate, 1 mM EDTA, pH 8.0). After migration, the gels were stained for 30 min in water containing ethidium bromide (2 μg/mL), according to standard procedures [23].

## 4. Conclusions

A series of arylidene-hydrazinyl-thiazole derivatives **2a**–**p** were synthesized with good yields by the Hantzsch protocol and their structures confirmed by NMR spectroscopy and mass spectrometry. The *in vitro* cytotoxicity was evaluated for all thiazoles **2a**–**p** on two carcinoma cell lines, MDA-MB231 and HELA. An excellent inhibition of cancer cells proliferation was reported for five thiazole derivatives. Among them, 2-(2-benzyliden-hydrazinyl)-4-methylthiazole **2a** exhibited a significant antiproliferative activity on both MDA-MB-231 (IC_50_: 3.92 µg/mL) and HeLa (IC_50_: 11.4 µg/mL) cell lines, while 2-[2-(4-methoxybenzylidene) hydrazinyl]-4-phenylthiazole **2e** showed a similar cytotoxic effect (IC_50_ value 11.1 µg/mL) on the HeLa cell line. It was also shown that these thiazole derivatives do not interact with plasmid DNA.
